# Mass Transfer of Anthocyanins during Extraction from Pre-Fermentative Grape Solids under Simulated Fermentation Conditions: Effect of Convective Conditions

**DOI:** 10.3390/molecules24010073

**Published:** 2018-12-26

**Authors:** Patrick C. Setford, David W. Jeffery, Paul R. Grbin, Richard A. Muhlack

**Affiliations:** Department of Wine and Food Science, School of Agriculture, Food and Wine, The University of Adelaide, PMB 1 Glen Osmond SA 5064, Australia; patrick.setford@adelaide.edu.au (P.C.S.); david.jeffery@adelaide.edu.au (D.W.J.); paul.grbin@adelaide.edu.au (P.R.G.)

**Keywords:** phenolic extraction, diffusion, anthocyanin, process modelling, wine colour, mass transfer

## Abstract

The colour of red wine is largely determined by the concentration of anthocyanins that are extracted from grape skins during fermentation. Because colour is a key parameter in determining the overall quality of the finished product, understanding the effect of processing variables on anthocyanin extraction is critical for producing a red wine with the desired sensorial characteristics. In this study, the effect of convective conditions (natural and forced) on the mass transfer properties of malvidin-3-glucoside (M3G) from pre-fermentative grape solids was explored at various liquid phase conditions representing stages of fermentation. A mathematical model that separates solid and liquid phase mass transfer parameters was applied to experimental extraction curves, and in all cases, provided a coefficient of determination exceeding 0.97. Calculated mass transfer coefficients indicated that under forced convective conditions, the extraction process was controlled by internal diffusion whereas under natural convection, both internal diffusion and liquid-phase mass transfer were relevant in determining the overall extraction rate. Predictive simulations of M3G extraction during active fermentation were accomplished by incorporating the current results with a previously developed fermentation model, providing insight into the effect of a dynamic liquid phase on anthocyanin extraction.

## 1. Introduction

Monomeric anthocyanins existing in pH-dependent equilibrium forms are among the largest class of phenolic compounds present in young red wines. In their cationic flavylium form, these compounds are directly responsible for the red colour in young red wines and over time interact with other (typically colourless) organic compounds, leading to long term stability of colour in older wines. As such, anthocyanins are regarded as one of the most important phenolic compounds responsible for the overall quality of red wine. During the red winemaking process, anthocyanins are extracted from the skins into the fermenting liquid through a multi-stage solid-liquid mass transfer process. This process first involves the diffusion of dissolved anthocyanins from within the grape skins to the solid-liquid boundary layer, followed by a liquid phase mass transfer step to the bulk of the fermenting liquid [1,2,3].

Following extraction, monomeric anthocyanins undergo a series of chemical and physical reactions, which include, but are not limited to, oxidation, copigmentation, self-association, adsorption to yeast lees, and re-adsorption to grape solids [3]. Due to the vast array of reactions and interactions, a distinct difficulty exists in accurately describing the extraction kinetics of anthocyanins and calculating kinetic coefficients during fermentative maceration. In previous years, phenolic extraction kinetics during red wine fermentation have been described using simple first- and second-order rate models, where a single term is used to describe the extractive phase and a second term is used to describe the subsequent reaction or degradation stage [4,5]. Due to the continuous evolution of ethanol during fermentative maceration, describing the extraction step of this process using a single term is a clear oversimplification that, although providing certain insights, is limited in providing quantitative predictions of future fermentative maceration scenarios. In addition, these models do not account for variability in the type, frequency, and duration of mixing operations employed during fermentative maceration—all of which would be expected to influence the overall rate of phenolic extraction [3,6]. 

During mixing operations, the rate of anthocyanin mass transfer from the solid-liquid boundary layer to the liquid bulk is a forced convective mass transfer process and can be well described with empirical correlations commonly employed in chemical engineering [7,8,9]. For the majority of red-wine maceration, however, the system is under natural convective conditions. To the authors’ knowledge, natural convective mass transfer of phenolic compounds—a critical step for furthering the understanding the extractive process of anthocyanins during winemaking—is as yet undescribed and external mass transfer parameters have not been quantified. 

Previously, internal diffusion rates of malvidin-3-*O*-β-d-glucoside (M3G), the predominant anthocyanin found in the skin of red wine grapes, have been quantified under various liquid-phase and industry-relevant temperature conditions [8]. The present study sought to extend upon this by evaluating the natural convective rates of external mass transfer of M3G in solutions emulating various stages of red wine fermentation, to simulate anthocyanin extraction under active fermentation scenarios.

## 2. Results and Discussion

### 2.1. Forced Convective Mass Transfer

To calculate the rate of natural convective mass transfer in the liquid phase, it was essential to first calculate the rate of internal mass transfer within the solid phase (grape skins). This was achieved using freshly pressed grape solids in solutions with varying concentrations of ethanol and sugar that were continuously stirred to achieve a well-mixed system. The rate of solute movement in the liquid phase could thereby be quantified using dimensionless numbers (described in Section 3.2). As such, the rate of external mass transfer could be separated from the overall extraction rate, allowing for solid phase mass transfer parameters at the varying liquid-phase conditions to be quantified (further details provided in Section 3.2 and Section 3.3). Three liquid-phase conditions representing the start (266 g L^−1^ sugar), middle (133 g L^−1^ sugar, 7% ethanol), and end point (14% ethanol) of a red-wine fermentation were chosen to determine the changes in mass transfer parameters over the course of a simulated fermentation.

**Figure 1:**
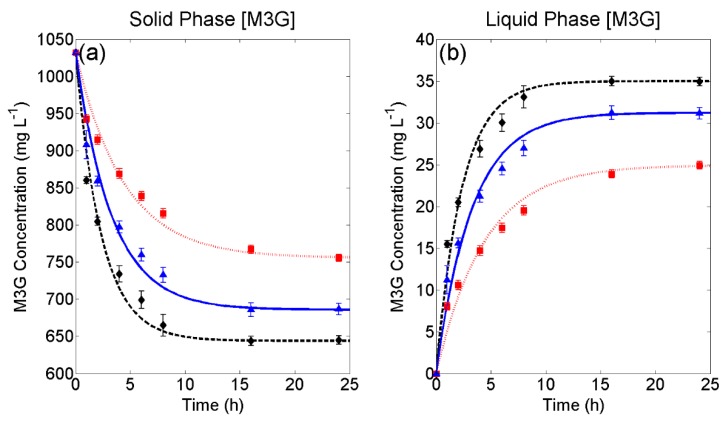
Experimental and fitted models under forced convection conditions for (**a**) solid phase depletion and (**b**) liquid phase accumulation of malvidin-3-glucoside. 266 g L^−1^ sugar; ▲, 133 g L^−1^ sugar, and 7% *v*/*v* ethanol; ◆, 14% *v*/*v* ethanol. Error bars represent the standard deviation across three replicates.

Figure 1 presents the experimental extraction curves of M3G from the grape solids (Figure 1a) and accumulation of M3G in the liquid phase (Figure 1b) at these varying conditions as well as the fit of Equations (14) and (15) of the forced convection model developed in Section 3.2 to the curves. Small error bars can be observed for each set of liquid phase conditions in Figure 1 (% RSD < 5.32, after the first hour of extraction), showing the high level of reproducibility among replicates. The root mean square error (RMSE) and R^2^ values were also calculated for each set of liquid phase conditions and in all cases, small RMSE (<1.58) and R^2^ values > 0.97 were observed (Table 1), indicating good agreement between experimental and mathematically-derived M3G concentrations. From Figure 1, it can be clearly observed that both the overall extraction rate (which in this case, can be approximated by the internal mass transfer rate) and maximum extractability of M3G are improved under conditions that emulate the later stages of fermentation (i.e., when more ethanol is produced). This is in agreement with other studies seeking to understand the influence of ethanol on phenolic extraction [8,10,11] and can be explained by the solvent properties of ethanol improving the solutions’ relative permittivity (the dielectric constant) [3], which may improve the rate of internal diffusion and extractability by aiding in the dissolution of M3G and in overcoming the liquids’ ability to penetrate the solid phase.

Table 1 presents a summary of mass transfer parameters for each set of forced convective experimental conditions, calculated according to the method outlined in Section 3.2. Here, the external diffusion coefficient ($D_{s\gamma }$) was calculated from the Wilke-Chang correlation (Equation (11)) and subsequently used to calculate the rate of external mass transfer ($k_{c\gamma }$) using a system of dimensionless values that describe fluid flow around particles (Equation (10)). From here, the rate of internal diffusion could be derived by fitting experimental data to Equations (14) and (15), and Equation (8) could then be used to calculate the internal diffusion coefficient. In each case, high rates of external mass transfer ($k_{c\gamma }$) were observed, indicating that the solid-liquid systems studied were indeed well-mixed. This is confirmed by the relatively high Biot numbers (>3.76 × 10^3^) calculated for each set of experimental conditions, where a value exceeding 10 would typically indicate that the overall extraction rate is controlled by internal diffusion [12]:

(1) $$Bi=\frac{k_{c\gamma }LK}{D_{s\beta }}$$

Although not the primary focus of this study, the use of grape berries for extraction experiments, which had been previously frozen, allowed for additional observation and comparison to be drawn regarding the effects of freezing on phenolic extraction. This is made possible as this study follows on directly from previous work [8] seeking to understand the relationship between liquid phase conditions and temperature on internal mass transfer parameters of M3G from fresh grape solids. For the same set of experimental conditions (266 g L^−1^ sugar and 14% *v*/*v* ethanol) and using the same parcel of berries as the previous study, we found distribution constant (K) values approximately three times larger with fresh berries [8] than those calculated in the present study using previously frozen berries at similar extraction temperature conditions. This result can be rationalised given that freezing causes the skin cells to burst, thus improving the fast leakage stage of extraction prior to solid-liquid diffusion [6]. A higher concentration of M3G in the juice would therefore occur due to leaking from broken cells upon crushing, lowering the available solid-phase M3G concentration at the onset of liquid contact, and thus decreasing the observed distribution constant. Similar results (in terms of rapid anthocyanin extraction into juice) have also been observed during flash release processing [13,14,15] and accentuated cut edge (ACE) maceration [16]. As such, the impact of pre-fermentative techniques, including must-freezing, flash détente, ultrasound, and skin size modification, on the initial leakage and overall distribution constant are important areas for future research, which, together with an understanding of mass transfer parameters developed in the present study, offer the potential to optimize extraction of M3G and other phenolic compounds relevant to wine quality. However, with the primary aim of this study being to explore the effect of convective conditions during extraction of anthocyanins, the use of previously frozen berries is not expected to impact on the conclusions drawn as the evaluation of external mass transfer parameters in the liquid phase are independent of internal mass transfer variables that may change as a result of grape freezing.

### 2.2. Natural Convective Mass Transfer

Natural convective mass transfer rates of M3G were evaluated in liquid solutions that were representative of different stages of red wine fermentation to better understand the extraction kinetics that would occur during the majority of a red wine fermentation (i.e., mixing is only conducted intermittently throughout the maceration period). This was accomplished by fitting Equations (14) and (15) to experimental data using the respective internal mass transfer coefficients ($k_{c\beta }$) that were evaluated during forced convective extractions (described in Section 2.1) and instead solving for the external mass transfer coefficients for each set of liquid phase conditions. 

**Figure 2:**
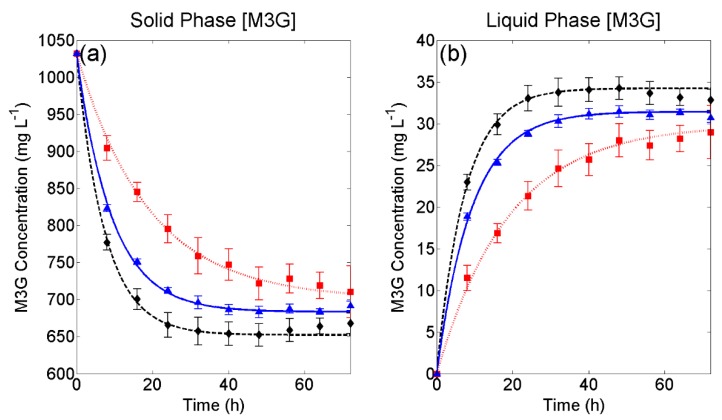
Experimental and fitted models under natural convection conditions for (**a**) solid phase depletion and (**b**) liquid phase accumulation of malvidin-3-glucoside. 266 g L^−1^ sugar; ▲, 133 g L^−1^ sugar and 7% *v*/*v* ethanol; ◆, 14% *v*/*v* ethanol. Error bars represent the standard deviation across three replicates.

Figure 2 presents the experimental extraction curves of M3G from the grape solids (Figure 2a) and accumulation of M3G in the liquid phase (Figure 2b) under natural convection as well as the fit of Equations (14) and (15) of the forced convection model developed in Section 3.2 to these curves for each set of liquid phase conditions. As before, small error bars can be observed (% RSD < 8.94), and in all cases, shows good experimental reproducibility between replicated extractions. Small RMSE values together with R^2^ values >0.97 in all cases indicates a good agreement between experimental and mathematically-derived M3G concentrations (Table 2) and that the model can be adequately used to describe M3G extraction under static liquid phase conditions. When compared to Figure 1, the overall rate of M3G extraction under natural convective conditions, as shown in Figure 2, is much slower than under forced convective conditions. This result can be explained, as the internal mass transfer rate of M3G is limited by the concentration at the solid-liquid interface and mixing minimises the concentration gradient between the solid-liquid boundary layer to the bulk of the liquid phase, creating a homogenous solution. As with forced convection, a higher overall extraction rate and maximum extractability can be seen with an increase in the extent of simulated fermentation (i.e., higher ethanol concentration, Figure 2).

Table 2 presents the mathematically derived mass transfer parameters for natural convective extraction of M3G at the liquid phase conditions investigated. Values for the internal mass transfer coefficient (solved in Section 2.1) are presented for comparison with the derived external mass transfer coefficients at natural convective conditions. For the liquid compositions representing mid-fermentation and the end of fermentation, similar values for the distribution constant were calculated for natural and forced convective conditions. This is rationalised due to the effect of forced convection, which should only affect the rate at which M3G is transferred from the solid-liquid interface to the bulk of the liquid and should not impact the maximum extractability. Calculated external mass transfer rates ($k_{c\gamma }$) under natural convective conditions were found to be lower in juice-like solutions than in wine-like solutions (Table 2). Because diffusivity is inversely proportional to the viscosity of the liquid [17], a likely explanation for this is the effect of sugar on the rheological properties of the liquid, whereby a higher viscosity caused by dissolved sugar would be expected to hinder mass transfer. Under natural convective conditions, the Biot numbers for M3G at various simulated fermentation stages were found to be between 7.83 × 10^−2^ and 1.13 × 10^−1^, indicating that mass transfer in the liquid phase has a strong influence on the overall rate of extraction. This result demonstrates the importance of both internal and external mass transfer to the overall rate of anthocyanin extraction in winemaking. Additionally, although previous mathematical models based on first- and second-order kinetics [4,5] may be adequately used to fit experimental extraction data during fermentation, the present work shows a more complex relationship, whereby knowledge of both solid and liquid phase mass transfer parameters is critical to informing extraction models with predictive capabilities.

### 2.3. Application in Simulated Wine Fermentations

Previous studies have highlighted a lack of understanding of the extraction mechanisms of phenolic compounds during fermentation, leading to a distinct difficulty in manipulating the phenolic content of finished wines [3,18]. Ultimately, the goal of modelling mass transfer under different conditions is the ability to predict phenolic extraction under various winemaking scenarios and thus close the previously highlighted gap. To further this knowledge, simulations of extraction scenarios for M3G under dynamic (continuously changing) liquid phase conditions were undertaken. This was accomplished by incorporating a previously developed wine fermentation model from Coleman et al. [19] (described in Section 3.4) with surface response models from Setford, Jeffery, Grbin, and Muhlack [8] for M3G that describe the distribution constant (Equation (2)) and the internal diffusion coefficient (Equation (3)), and calculating the rate of internal mass transfer (Equation (8)) as functions of the liquid phase conditions:

(2) $$\begin{array}{l} K=9.97\;\times \;10^{-2}\;+\;3.33\;\times \;10^{-2}T+1.04\;\times \;10^{-2}C_{g}+3.74\;\times \;10^{-2}C_{EtOH}\\ \;+1.23\;\times \;10^{-2}TC_{g}-9.64\;\times \;10^{-3}TC_{EtOH}\\ \;+7.91\;\times \;10^{-3}C_{g}C_{EtOH}+9.86\;\times 10^{-3}TC_{g}C_{EtOH} \end{array}$$

(3) $$D_{s\beta }\;=\;2.75\;\times \;10^{-13}\;+\;2.11\times 10^{-13}T+1.09\times 10^{-16}C_{EtOH}+8.52\times 10^{-14}TC_{EtOH}$$

Further extending these predictive models, a second-degree polynomial was fitted to the experimentally determined values of external mass transfer coefficients for natural convective mass transfer presented in Table 2, allowing the prediction of $k_{c\gamma }$ as a function of the relative extent of fermentation:

(4) $$k_{c\gamma }=-4.94\times 10^{-9}Ex^{2}+8.27\times 10^{-9}Ex+2.20\times 10^{-9}$$

where Ex represents the extent of liquid phase fermentation (i.e., 266 g L^−1^ sugar, 0% *v*/*v* ethanol = 0, 0 g L^−1^ sugar, 14% *v*/*v* ethanol = 1). The values for K, $k_{c\beta }$, and $k_{c\gamma }$ calculated using this method were then used to solve Equations (14) and (15) and generate extraction curves with continuously changing mass transfer variables resulting from changes in liquid phase concentrations of sugar and ethanol.

**Figure 3:**
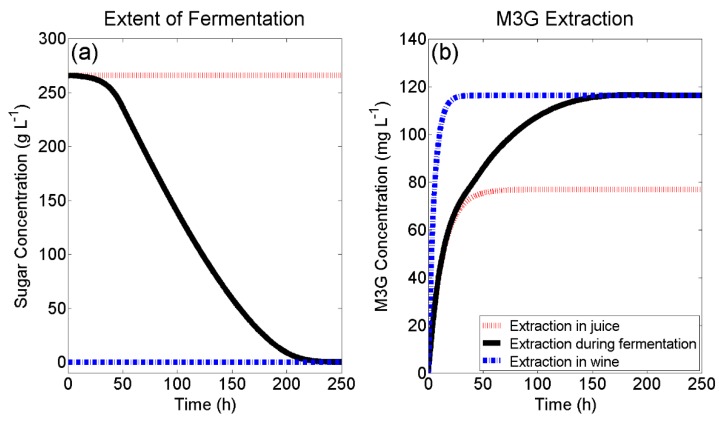
(**a**) Simulated red wine fermentation kinetics and (**b**) the resulting simulated extraction kinetics of M3G. Red and blue lines indicate the rate and extent of M3G extraction that would occur under constant liquid phase conditions for juice and wine, respectively.

Figure 3a presents a typical red wine fermentation curve at 20 °C (black curve), solved using the fourth order Runge-Kutta method in MATLAB according to the method outlined in Section 3.4 and using the following set of initial conditions: T = 20 °C $\mu_{0}$ = 0.05 h^−1^, $k_{d,0}$ = 0.0001 h^−1^, $B_{0}$ = 0.05 g_EtOH_ g_biomass_ h^−1^, $X_{0}$ = $X_{A,0}$ = 0.05 g L^−1^, $N_{0}$ = 0.08 g L^−1^, $E_{0}$ = 0 g L^−1^, $S_{0}$ = 266 g L^−1^. Evidently, M3G extraction is initially limited by the fermentation lag-phase, where the liquid phase contains a high concentration of sugar and no ethanol. Although experimental extractions of M3G under fixed liquid phase and natural convective conditions reached a maximum liquid phase concentration within 72 h (Figure 2b), the simulated extraction curve presented in Figure 3b supported literature observations of extraction during active fermentation, where the time taken to reach a maximum anthocyanin concentration was seen to be considerably longer (up to a week) than extraction under fixed solvent conditions [4,5,20]. Figure 3b highlights the importance of ethanol in maximising extracted anthocyanins and implies that pre-fermentative maceration techniques, such as cold-soaking, in the absence of ethanol would not be expected to improve the concentration of M3G at the end of fermentation.

**Figure 4:**
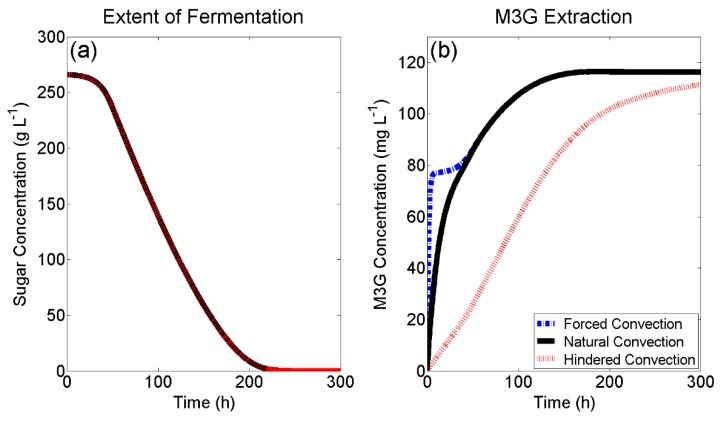
(**a**) Simulated red wine fermentation kinetics and (**b**) the resulting simulated extraction kinetics of M3G under different modes of liquid phase convection.

Figure 4a shows overlapping fermentation curves for the three simulated M3G extractions in Figure 4b. This is because the same initial conditions were used to solve for the fermentation model (described in Section 3.4), which does not consider liquid phase mass transfer as a fermentation parameter. In Figure 4b, ‘Forced Convection’ was modelled using an external mass transfer coefficient of 1 × 10^−4^ (Biot number > 1000), ‘Natural Convection’ was modelled using a varying external mass transfer coefficient according to Equation (4), and ‘Hindered Convection’ was modelled using a fixed system Biot number of 0.01 to show the effect of further limiting the rate of external mass transfer.

Under forced convective conditions, where internal mass transfer is the rate-controlling step for extraction, a fast initial extraction within the first few hours of fermentation can be observed followed by a lag in the extraction (Figure 4b) that follows the fermentation lag period (Figure 4a) due to a lack of ethanol in the liquid phase. Following this, M3G extraction is limited by the concentration of ethanol in the system, which increases the distribution constant. Under natural convective conditions, M3G extraction appears to follow a simple first-order rate curve—a commonly used model for anthocyanin extraction during fermentation [4,5].

The initial extractive phase for M3G is slower than with forced convection due to the lower rate of external mass transfer, which in turn means the overall extraction rate is dependent on both internal and external mass transfer properties. Despite this, a similar observation to forced convective conditions can be seen (Figure 4b), whereby after the initial fermentation lag phase (Figure 4a), the rate of M3G extraction is limited by the concentration of ethanol in the system. This implies that in an evenly distributed natural convective system, the same maximum M3G concentration can be achieved as in a well-mixed system, due to the requirement of ethanol in the liquid phase to increase the distribution constant (K) and maximise the M3G concentration. A simulation of ‘Hindered Convection’ was selected to show the case where normal natural convective extraction may be slowed, such as within the “cap” (grape skins, seeds, stems) of an active red wine fermentation. In this case, a solute particle (such as M3G) within the centre of the cap would need to diffuse through the tortuous path around the solids to the boundary layer separating the cap and the bulk of the liquid phase. Under these conditions, an initial lag in liquid phase M3G concentration would be expected, as a lower external mass transfer coefficient decreases the rate at which M3G is moved from the solid-liquid boundary layer to the liquid bulk, which in-turn lowers the M3G concentration gradient within the grape skin. From Figure 4b, it can also be observed that under hindered convective conditions (Bi = 0.01), ethanol production is no longer the rate limiting step for M3G extraction, as was the case for both forced and natural convection (after the initial fermentation lag phase), and that the rate of liquid phase mass transfer is limiting.

## 3. Materials and Methods 

### 3.1. Experimental

#### 3.1.1. Experimental Design

For each of the forced and natural convective conditions, three different liquid solutions differing in concentrations of sugar (glucose), ethanol, and water were chosen to emulate extractive conditions representative of the start, middle, and end of a red wine fermentation process for a wine finishing at 14% *v*/*v* of ethanol. Extractions were undertaken in a temperature controlled room nominally set at an ambient temperature of 20 °C. These experimental conditions are presented in Table 1 and Table 2. Liquid solutions were prepared and allowed to equilibrate to the ambient air temperature 48 h prior to the commencement of extraction. The local temperature around the extractions was monitored by way of a temperature data logger (model XC0424, Jaycar Electronics, Adelaide, Australia) and was observed to average at 20.2 °C throughout the extraction period. The pH values of liquid phases after one hour of extraction were found to be between 3.81 and 4.16 and finished between 3.64 and 4.03. This variation in pH is not expected to have had an impact on the extraction results as previous work [21] on polyphenol extraction from grape wastes found comparable anthocyanin extraction yields within the pH range of 2 to 8.66 in hydro-alcoholic solutions of 0% and 25% ethanol.

#### 3.1.2. Sample Preparation

Merlot grapes from the University of Adelaide’s Coombe vineyard were hand harvested (total soluble solids of 14.3 °Bé, pH 3.6, titratable acidity of 3.9 g L^−1^) on 24 February 2016 and were frozen at −20 °C until processing. Prior to crushing, berries were thawed overnight at an ambient temperature of 10 °C and destemmed by hand while still cool. In total, 11.02 kg of randomly sampled berries were then crushed by hand using a stainless steel plunger until all berries were visibly crushed. The must was pressed using a 4.4 L hand operated stainless steel basket press to separate the solids (skins and seeds) from liquid juice. The total mass of pressed solids was 4.58 kg and the collected juice volume was 5.70 L.

#### 3.1.3. Extraction Procedure

For each extraction, 150 g of pressed solids were added to 2 L extraction vessels prior to the addition of 1.5 L of liquid solution to begin the extraction process. Following liquid addition, lids containing a 1 cm diameter hole to allow the insertion of an overhead mechanical stirrer were fitted to the extraction vessels. For the extractions conducted under forced convective conditions, the contents of the vessels were continually stirred throughout the extraction period at a rotational speed of approximately 300 rpm to allow for the calculation of mass transfer parameters within the solid phase. For the extractions conducted under natural convective conditions, the overhead stirrers remained off throughout the extraction with mixing only occurring for 5 s immediately prior to sample collection to ensure a homogenous liquid phase representative of the entire mixture. All extractions were conducted in triplicate. For each set of conditions, 10 mL samples were collected throughout the extraction process until an equilibrium was reached. For the trials under forced convective conditions, samples were collected more frequently within the first 8 h of the extraction to produce appropriate concentration curves. After collection, samples were immediately centrifuged and kept at −20 °C until analysis by HPLC.

#### 3.1.4. Quantification of Malvidin-3-glucoside in Extracts

Samples taken throughout the extraction period were thawed at ambient temperature and sonicated using a benchtop sonicator (model FXP10M, Unisonics, Sydney, Australia) at 50 Hz for 10 min to dissolve precipitated solids. These samples were then centrifuged at 10,000 rcf and 1.5 mL of supernatant transferred to amber HPLC vials for analysis by HPLC using the method described by Setford, Jeffery, Grbin, and Muhlack [1]. 

#### 3.1.5. Quantification of Malvidin-3-glucoside in Solids

Freshly pressed grape solids (250 g) used in the extraction trials were homogenised using a Grindomix GM200 homogeniser (Retsch, Haan, Germany) for 20 s at a speed of 8000 rpm. The homogenate was then well mixed by hand and 1 g samples of the mixture were transferred to 10 mL centrifuge tubes, in triplicate. To each tube, 10 mL of 50% *v*/*v* aqueous ethanol was added and the mixture inverted by hand at 10 min intervals for a one hour extraction period. The mixtures were then centrifuged at 3220 rcf for 10 min and the supernatant decanted to a new centrifuge tube. These extracts were kept at −20 °C until analysis by HPLC as described in Section 3.1.4. Thawed samples were diluted to 12.5% *v/v* aqueous ethanol with water prior to injection. These initial M3G concentrations were used to calculate all other solid-phase M3G concentrations though a mass balance of the extraction system based on the respective liquid-phase samples collected throughout the experiment.

#### 3.1.6. Determination of Physical Parameters

A Viscoball Höppler viscometer (Fungilab, Barcelona, Spain) was used to measure the dynamic viscosity of the extraction solutions, at the same temperature of 20 °C as used during the extraction experiment. Temperature was maintained by circulating cooling water through the outer shell of the viscometer, with cooling water maintained at 20 °C via an external water bath (model TBC-3-22, Thermoline L + M, Smithfield, NSW, Australia) with active temperature control (controller model TBC-TU4, Thermoline L + M, Smithfield, NSW, Australia). Cameras (model KYT-U130-01MBWCS, Kayeton Technology Company Ltd., Shenzhen, China) at a frame rate of 30 frames per second were used to monitor the start and end points of the viscometer to increase the precision of time measurements. Aspen HYSYS software (Aspen Technology Inc, Bedford, MA, USA) was used to calculate the density at each set of liquid phase conditions based on the liquid phase composition. The solvent molecular mass and association parameter were calculated by the sum of individual ethanol, sugar, and water concentrations in the liquid phase (Equations (12) and (13), respectively).

### 3.2. Forced Convection Model

The extraction of anthocyanins during fermentative maceration is a solid-liquid mass transfer process controlled by diffusion after the initial leakage around broken skin cells [16,22]. This process consists of three steps commonly grouped together and known as internal diffusion, where the liquid first penetrates the solid to the location of the solute, dissolves the solute, and finally diffuses from within the solid to the solid-liquid boundary layer. At this point, the dissolved solute is then transferred into the bulk of the liquid either by liquid-phase diffusion in the case of natural convection (where no external force is applied to the liquid phase), or by forced convective mass transfer as would be observed during mixing operations. Due to the waxy outer layer of grape skins, diffusion is considered only to take place radially inwards, perpendicular to the surface of the grape skin. A mechanistic model for this process taking into account solute diffusion within the solid, solute mass transfer from the interface to the liquid bulk, and an equilibrium relationship between solid and liquid concentrations can be summarised as follows [8,23,24]:

(5) $$\left(1-\varepsilon \right)\frac{dc_{\beta }}{dt}=k_{c\beta }a\left(c_{\beta i}-c_{\beta }\right)$$

(6) $$\varepsilon \frac{dc_{\gamma }}{dt}=k_{c\gamma }a\left(c_{\gamma i}-c_{\gamma }\right)$$

(7) $$c_{\gamma i}=Kc_{\beta i}$$

Here, the rate of internal diffusion within the solid phase ($D_{s\beta }$) can be determined through a correlation developed in Setford, Jeffery, Grbin, and Muhlack [8] for one-dimensional mass transfer across a plane:

(8) $$D_{s\beta }=\frac{k_{c\beta }4L}{\left(1-\varepsilon \right)\pi^{2}}$$

Under forced convective conditions, the rate of external mass transfer can be determined through a correlation of dimensionless values describing the physical properties of the system:

(9) Sh=2+0.95Re12Sc13

where the Reynolds number (Re) can be calculated as a function of the revolutionary speed (N), in revolutions per second, and mixer diameter (D) and:

(10) $$Re=\frac{nD^{2}\rho_{\gamma }}{\mu_{\gamma }},\;Sh=\frac{k_{c\gamma }L}{D_{s\gamma }},\;Sc=\frac{\eta }{\rho_{\gamma }D_{s\gamma }}$$

The rate of liquid phase diffusion necessary for calculating the Sherwood (Sh) and Schmidt (Sc) numbers can be determined from the Wilke-Chang correlation:

(11) $$D_{s\gamma }=\frac{1.173\times 10^{-16}{\left(\varphi M_{\gamma }\right)}^{1/2}T}{\eta V_{A}^{0.6}}$$

An additive method described by Geankoplis [9] based on the chemical structure of M3G is then used to determine the volume of the solute ($V_{A}$), and the average liquid phase association factors and molecular mass can be determined from the mole fractions of the major components of the liquid phase:

(12) $$M_{\gamma }=x_{EtOH}M_{EtOH}+x_{water}M_{water}+x_{Glucose}M_{Glucose}$$

(13) $$\varphi =x_{EtOH}\varphi_{EtOH}+x_{water}\varphi_{water}+x_{Glucose}\varphi_{Glucose}$$

An analytical solution to Equations (5) to (7) that describes the average solute concentration in both phases (first proposed by Espinoza-Pérez, Vargas, Robles-Olvera, Rodríguez-Jimenes, and García-Alvarado [23]) yielded the following system of equations:

(14) $$c_{\beta }=c_{\beta 0}\left(C_{1}e^{r_{1}t}+C_{2}e^{r_{2}t}\right)$$

(15) $$c_{\gamma }=c_{\beta 0}\left(C_{3}e^{r_{1}t}+C_{4}e^{r_{2}t}\right)$$

where:

$$r_{1,2}=-\frac{b_{1}+b_{2}}{2}\pm \frac{\sqrt{{\left(b_{1}+b_{2}\right)}^{2}-4\left(b_{1}b_{2}-b_{3}b_{4}\right)}}{2}$$

$$C_{1}=\frac{r_{1}+b_{1}}{r_{1}-r_{2}},\;C_{2}=\frac{r_{2}+b_{1}}{r_{2}-r_{1}},\;C_{3}=\frac{b_{3}}{r_{1}-r_{2}},\;C_{4}=\frac{b_{3}}{r_{2}-r_{1}}$$

$$b_{1}=\frac{k_{c\gamma }a\left(1-\psi_{1}\right)}{\varepsilon },\;b_{2}=\frac{k_{c\beta }a\left(1-\frac{\psi_{2}}{K}\right)}{\left(1-\varepsilon \right)},\;b_{3}=\frac{k_{c\gamma }a\psi_{2}}{\varepsilon },\;b_{4}=\frac{k_{c\beta }a\frac{\psi_{1}}{K}}{\left(1-\varepsilon \right)}$$

$$\psi_{1}=\frac{1}{1+\frac{k_{c\beta }}{Kk_{c\gamma }}},\;\psi_{2}=\frac{k_{c\beta }/k_{c\gamma }}{1+\frac{k_{c\beta }}{Kk_{c\gamma }}}$$

To determine the rate of internal mass transfer, $k_{c\beta }$, MATLAB software (version R2013a) was used to fit non-linear regressions to Equations (14) and (15) using experimentally determined M3G concentrations, whereby the residuals of the model are minimised by adjusting $k_{c\beta }$. Table 3 presents a summary of the physical parameters and system variables used for the model solution.

### 3.3. Natural Convection Model

Because the rate of internal diffusion and mass transfer within the solid phase is independent of the mode of convection [23,24], the internal mass transfer coefficients ($k_{c\beta }$) for natural convective trials are taken as those solved under forced convective conditions at the respective temperature and solvent conditions. The external mass transfer coefficient ($k_{c\gamma }$) for natural convective trials was then obtained through a non-linear regression of Equations (14) and (15) whereby the residuals of the model solution were set to be minimised.

### 3.4. Simulating Extraction during Fermentation 

To simulate M3G extraction under active fermentation scenarios, a predictive wine fermentation model originally proposed in Cramer et al. [26] and later modified in Coleman, Fish, and Block [19] was used, which consists of five coupled ordinary differential equations (ODEs):

(16) $$\frac{dX}{dt}=\mu X_{A}$$

(17) $$\frac{dX_{A}}{dt}=\mu X_{A}-k_{d}X_{A}$$

(18) $$\frac{dN}{dt}=-\frac{\mu X_{A}}{Y_{X/N}}$$

(19) $$\frac{dE}{dt}=BX_{A}$$

(20) $$\frac{dS}{dt}=-\frac{BX_{A}}{Y_{E/S}}$$

Here, X is the yeast biomass concentration, $X_{A}$ is the active yeast concentration, N is the nitrogen concentration (which is the yeast-growth limiting nutrient in the model), E is the ethanol concentration, and S is the sugar concentration. The specific yeast cell growth rate and sugar consumption rate are described by Michaelis-Menten kinetics:

(21) $$\mu =\frac{\mu_{max}N}{K_{N}+N}$$

(22) $$B=\frac{B_{max}S}{K_{S}+S}$$

where $\mu_{max}$ is the maximum yeast growth rate, $K_{N}$ is the Monod constant for nitrogen consumption, $B_{max}$ is the maximum sugar consumption rate, and $K_{S}$ is the constant for sugar consumption. The yeast cell death rate is defined as a function of the ethanol concentration:

(23) $$k_{d}=k_{d}^{\prime }E$$

where $k_{d}^{\prime }$ denotes the sensitivity of yeast cells to ethanol. To solve this system of ODEs describing fermentation kinetics, a series of temperature-dependent multi-linear models described by Coleman, Fish, and Block [19] were used to estimate the values of parameters within Equations (16) to (23). A summary of the coefficients used for each parameter is provided in Table 4. 

### 3.5. Statistical Analysis

For each set of experimental conditions, the efficacy of the proposed model’s fit was determined based on two parameters: The root mean square error (RMSE) and the coefficient of determination ($R^{2}$). These values were determined by:

(24) $$RMSE=\sqrt{\frac{1}{N}\overset{N}{\underset{i=1}{\sum }}{\left(c_{\gamma ,pred,i}-c_{\gamma ,exp,i}\right)}^{2}}$$

(25) $$R^{2}=1-\frac{\sum_{i=1}^{N}{\left(c_{\gamma ,pred,i}-c_{\gamma ,exp,i}\right)}^{2}}{\sum_{i=1}^{N}{\left(c_{\gamma ,pred,i}-{\bar{c}}_{\gamma ,exp}\right)}^{2}}$$

Here, $c_{\gamma ,exp,i}$ is the experimentally determined M3G liquid phase concentration, $c_{\gamma ,pred,i}$ is the concentration predicted by the model, ${\bar{c}}_{\gamma ,exp}$ is the replicate mean of experimentally-determined concentrations in the liquid phase, and N is the number of replicates for each trial.

## 4. Conclusions

The extraction of M3G from pre-fermentative red grape solids under conditions emulating different stages of fermentation with respect to sugar and ethanol concentrations were explored under natural and forced convective conditions. A previously developed mathematical model based on first principles and a series of empirical correlations were used to describe the solid-liquid extraction process and calculate relevant mass transfer parameters at all experimental conditions. Calculated Biot numbers indicate that under forced convection, the process was controlled by internal diffusion, whereas under natural convection, both internal diffusion and liquid-phase convection controlled the extraction rate. Predictive simulations of extraction under fermentation conditions (continuously evolving liquid phase) showed that under natural convective conditions, the extraction of M3G was also strongly influenced by the rate of ethanol evolution. This observation of a more complex extraction relationship, which includes the influence of ethanol, provides insight into other studies comparing the effectiveness of mixing technologies during fermentation, where conflicting results have previously been observed.

## Figures and Tables

**Table 1:** Summary of liquid phase conditions, mass transfer properties ($D_{s\gamma }$, $k_{c\gamma }$, $D_{s\beta }$, $k_{c\beta }$, and K), Biot number (Bi), and statistical parameters (RMSE and $R^{2}$) for malvidin-3-glucoside solved using the method outlined in Section 3.2 for forced convective extraction.

	Liquid Phase Conditions		
Property or Mass Transfer Variable	Juice	Mid-Ferment	Wine
Sugar (g L^−1^)	266	133	0
Ethanol (% *v*/*v*)	0	7	14
$D_{s\gamma }$ (m^2^ s^−1^)	4.22 × 10^−12^	5.01 × 10^−12^	5.66 × 10^−12^
$k_{c\gamma }$ (m s^−1^)	1.70 × 10^−4^	1.94 × 10^−4^	2.16 × 10^−4^
$D_{s\beta }$ (m^2^ s^−1^)	2.09 × 10^−13^	3.49 × 10^−13^	5.47 × 10^−13^
$k_{c\beta }$ (m s^−1^)	2.45 × 10^−10^	4.10 × 10^−10^	6.41 × 10^−10^
K	3.30 × 10^−2^	4.55 × 10^−2^	5.43 × 10^−2^
Bi	4.71 × 10^3^	4.43 × 10^3^	3.76 × 10^3^
RMSE	1.33	1.44	1.58
$R^{2}$	0.972	0.972	0.980

**Table 2:** Summary of liquid phase conditions, mass transfer properties ($D_{s\gamma }$, $k_{c\gamma }$, $D_{s\beta }$, $k_{c\beta }$, and K), Biot number (Bi), and statistical parameters (RMSE and $R^{2}$) for malvidin-3-glucoside solved using the method outlined in Section 3.2 for natural convective extraction.

	Liquid Phase Conditions		
Mass Transfer Variable	Juice	Mid-Ferment	Wine
Sugar (g L^−1^)	266	133	0
Ethanol (% *v*/*v*)	0	7	14
K	4.26 × 10^−2^	4.60 × 10^−2^	5.25 × 10^−2^
$k_{c\beta }$ (m s^−1^)	2.45 × 10^−10^	4.10 × 10^−10^	6.41 × 10^−10^
$k_{c\gamma }$ (m s^−1^)	2.20 × 10^−9^	4.93 × 10^−9^	5.53 × 10^−9^
Bi	7.83 × 10^−2^	1.13 × 10^−1^	9.23 × 1^−2^
RMSE	0.58	0.59	1.61
$R^{2}$	0.996	0.995	0.970

**Table 3:** Summary of physical properties and shape variables of the experimental extraction system.

Property or Shape Variable	Value	Source
a (m^2^ m^−3^)	5747	Mathematically derived
ε	0.9173	Experimentally determined
$c_{\beta 0}$ (kg m^−3^)	1.032	Experimentally determined
L (m)	1.74 × 10^−4^	Jin, et al. [25]
$V_{A}$ (L mol^−1^)	0.5259	Geankoplis [9]
η (cP)	Varied	Experimentally determined
$\rho_{\gamma }$ (kg m^−3^)	Varied	HYSYS (Hysys, Operations Guide., 2005)
$M_{\gamma }$ (g mol^−1^)	Varied	Equation (12)
φ	Varied	Equation (13)

**Table 4:** Coefficients for estimating fermentation model parameters as a function of temperature, where: $Parameter=a_{0}+a_{1}T+a_{2}T^{2}$.

	Coefficients for Regression Models		
Parameter	$a_{0}$	$a_{1}$	$a_{2}$
$log\left(\mu_{max}\right)$	−3.92	7.82 × 10^−2^	-
$log\left(K_{N}\right)$	−4.73	-	-
$log\left(k_{d}^{\prime }\right)$	−9.81	−1.08 × 10^−3^	4.78 × 10^−3^
$log\left(Y_{X/N}\right)$	3.50	−3.61	-
$log\left(Y_{E/S}\right)$	−5.98 × 10^−1^	-	-
$log\left(B_{max}\right)$	−2.30	7.71 × 10^−2^	-
$log\left(K_{S}\right)$	2.33	-	-

## Abbreviations

**Nomenclature**	
a	Specific surface area for mass transfer (m^2^ m^−3^)
$b_{1},\ldots ,b_{4}$	Constants required to solve Equations (14) and (15)
B	Rate of sugar consumption per cell (g_EtOH_ g_biomass_ h^−1^)
$B_{max}$	Maximum sugar consumption rate (g_EtOH_ g_biomass_ h^−1^)
c	Phenolic compound concentration (mg L^−1^)
$C_{1},\ldots ,C_{4}$	Constants required to solve Equations (14) and (15)
$C_{EtOH}$	Ethanol concentration (% *v/v*)
$C_{g}$	Glucose concentration (g L^−1^)
D	Mechanical stirrer diameter (m)
$D_{s}$	Solute (s) mass diffusivity (m^2^ s^−1^)
$k_{c}$	Mass transfer coefficient (m s^−1^)
$k_{d}$	Yeast cell death rate (h^−1^)
$k_{d}^{\prime }$	Coefficient describing yeast sensitivity to ethanol
K	Distribution constant
$K_{N}$	Constant for nitrogen-limited yeast growth
$K_{S}$	Constant for sugar consumption by yeast
L	Characteristic length for diffusive mass transfer (m)
M	Molar mass (g mol^−1^)
n	Stirrer speed (rpm)
N	Nitrogen concentration (g L^−1^)
$r_{1,2}$	Constants required to solve Equations (14) and (15)
$R^{2}$	Coefficient of determination
RMSE	Root mean square error
S	Sugar concentration (g L^−1^)
t	Time (s)
T	Temperature (°C)
$V_{A}$	Solute molar volume (L mol^−1^)
X	Yeast biomass concentration (g L^−1^)
$X_{A}$	Active yeast biomass concentration (g L^−1^)
$Y_{E/S}$	Yield coefficient for ethanol production to sugar consumption
$Y_{X/N}$	Yield coefficient for cell growth to nitrogen consumption
**Dimensionless groups**	
Bi	Biot number
Re	Reynolds number
Sc	Schmidt number
Sh	Sherwood number
**Greek symbols**	
ε	Solvent volume fraction
φ	Association parameter
η	Dynamic viscosity (cP)
μ	Specific yeast cell growth rate (s^−1^)
$\mu_{max}$	Maximum specific yeast cell growth rate (s^−1^)
ρ	Density (kg m^−3^)
$\psi_{1,2}$	Constants required to solve Equations (14) and (15)
**Subscripts**	
0	At the initial or reference point
exp	Experimentally obtained
i	At the solid-liquid interface
pred	Predicted by model
β	Solid phase
γ	Liquid phase
